# Assessment of Potential Toxicity of Hyaluronic Acid-Coated Magnetic Nanoparticles on Maize (*Zea mays*) at Early Development Stages

**DOI:** 10.3390/molecules30061316

**Published:** 2025-03-14

**Authors:** Mihaela Răcuciu, Cristina-Nicoleta Precup, Maria Denisa Cocîrlea, Simona Oancea

**Affiliations:** 1 Environmental Sciences and Physics Department, Faculty of Sciences, Lucian Blaga University of Sibiu, 5-7 Dr. I. Ratiu Street, 550012 Sibiu, Romania; cristinanicoleta.precup@ulbsibiu.ro; 2 Agricultural Sciences and Food Engineering Department, Lucian Blaga University of Sibiu, 7-9 Dr. I. Ratiu Street, 550024 Sibiu, Romania; denisa.cocirlea@ulbsibiu.ro

**Keywords:** iron oxide nanoparticle, hyaluronic acid, maize, plant growth, enzymatic activity, chromosomal aberrations

## Abstract

The effectiveness of iron oxide nanoparticles in enhancing crop plant development depends on their stabilization. In this study, the effect of hyaluronic acid (HA), used both as a stabilizer for iron oxide nanoparticles (HA-MNP) and independently, was evaluated in maize seedlings. Different concentrations of HA-MNP (0.625–7.5 mg/L) were tested alongside a 0.01% HA solution. Growth parameters, antioxidant enzyme activities (peroxidase and polyphenol oxidase), photosynthetic pigments (chlorophyll and carotenoids), phenolic content, and genotoxicity were analyzed. While HA alone led to slight decreases in seedling length, pigment content, and polyphenol levels compared to the control, it increased peroxidase activity and mitotic index. Lower concentrations of HA-MNP (below 2.5 mg/L) enhanced seedling growth, likely due to improved iron uptake, whereas higher concentrations reduced pigment and phenolic content. All HA-MNP concentrations induced genotoxic effects, which was proven by an increased mitotic index and chromosomal aberrations, indicating both positive and defensive plant responses to oxidative stress. These findings suggest a complex interaction between HA, HA-MNP, and maize seedlings, where HA concentrations play a significant role in modulating growth and stress response, while higher concentrations may induce toxicity.

## 1. Introduction

The development of nanomaterials has significantly contributed to advancements in agricultural biotechnology. Among these, iron oxide nanoparticles have attracted considerable attention owing to their unique physical–chemical properties, including high surface-area-to-volume ratio, superparamagnetic behavior, and enhanced reactivity. These characteristics distinguish them from their bulk counterparts, making them valuable for various plant-related applications [1]. Studies have shown that iron oxide nanoparticles can positively influence plant growth, development, and physiology by enhancing iron bioavailability, which is crucial for chlorophyll synthesis and overall plant health [2,3,4]. Increased iron uptake facilitated by these nanoparticles can lead to improved photosynthetic efficiency and higher biomass accumulation [5]. Furthermore, iron oxide nanoparticles have been reported to promote seed germination and root elongation, thereby improving plant establishment [6]. Additionally, they may enhance plant tolerance to abiotic stressors such as drought, salinity, and heavy metal contamination [7].

Despite these benefits, the effects of iron oxide nanoparticles on plants are highly dependent on their concentration, exposure duration, and plant species. At elevated concentrations, they have the potential to induce oxidative stress, disrupt cellular processes, and cause phytotoxicity, owing to the overproduction of reactive oxygen species (ROS) [8,9]. This can lead to membrane damage, impaired metabolic functions, and growth inhibition. The threshold concentration at which beneficial effects are surpassed by harmful effects varies among plant species and is influenced by environmental conditions, growth stage, and nanoparticle properties. Several nanoparticle characteristics, such as size, shape, and surface modifications, play critical roles in determining their interactions with plant tissues, as reported in the literature [10,11]. Smaller nanoparticles generally have greater penetration ability and can more efficiently translocate within plant systems, whereas surface modifications influence stability, reactivity, and biocompatibility [12]. The use of stabilizing agents can enhance nanoparticle dispersion, reduce agglomeration, and minimize toxicity, ensuring more controlled interaction with biological systems [13,14,15].

Among the various stabilizing agents, hyaluronic acid (HA) has gained attention owing to its biodegradability, low toxicity, and biocompatibility [16]. HA is a naturally occurring polysaccharide with a high molecular weight and linear structure that is widely used in biomedical applications, such as tissue engineering, cancer therapy, and drug delivery [17,18,19]. However, its potential role in modulating nanoparticle–plant interactions remains unexplored. Given their ability to improve nanoparticle stability and reduce aggregation, HA-stabilized iron oxide nanoparticles (HA-MNPs) could present novel opportunities in plant science and agriculture.

In this study, we investigated the genotoxic and phytotoxic effects of hyaluronic acid (HA)-stabilized iron oxide/magnetic nanoparticles (HA-MNP) on maize (*Zea mays*) seedlings during early growth stages. We evaluated the effect of HA-MNP at concentrations ranging from 0.625 to 7.5 mg/L on plant growth parameters (stem and root length), chlorophyll content, total phenolic content, enzymatic activity (peroxidase and polyphenol oxidase), and genotoxicity (mitotic index and chromosomal aberrations). By analyzing these effects, our research aims to contribute to a better understanding of the role of HA-stabilized nanoparticles in plant development and provide insights into their safe and sustainable integration into agricultural ecosystems.

## 2. Results

### 2.1. HA-MNP Phytotoxicity on Maize

Experimental samples treated with increasing concentrations of HA-MNP demonstrated a progressive increase in the germination percentage. Notably, the samples exposed to 1.25 mg/L, 1.875 mg/L, and 2.5 mg/L HA-MNP achieved a level of 100% germination, surpassing the 96% germination rate of the control (untreated samples). Seedlings subjected to daily HA-MNP treatment exhibited no significant alterations in root moisture content. However, a statistically significant reduction of approximately 6% was observed in the stem moisture content at the highest applied concentration (7.5 mg/L).

**Figure 1:**
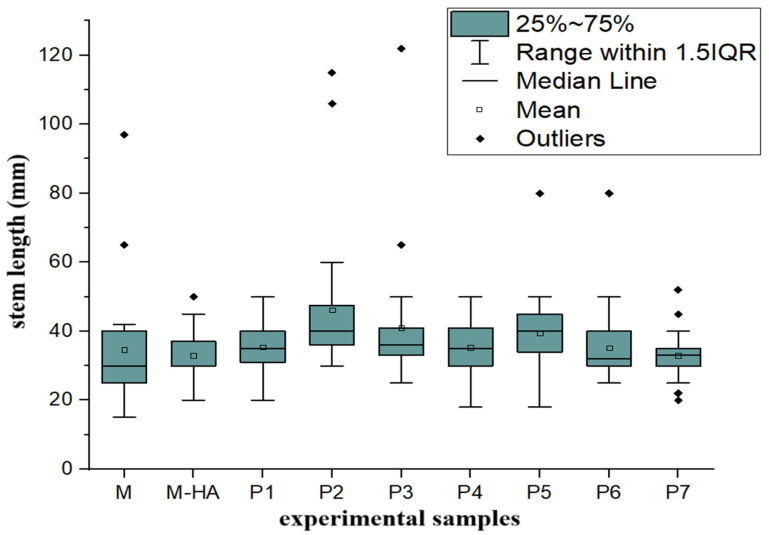
Box plot representation of the stem lengths of seedlings developed in the presence of HA-MNP nanoparticles at different concentrations. M, control group treated with distilled water; M-HA, control group treated with 0.01% hyaluronic acid solution. P1 to P7 represent samples treated with different concentrations of HA-MNP: 0.625–1.25–1.875–2.5–3.125–3.75–7.5 mg/L (according to Table 2).

**Figure 2:**
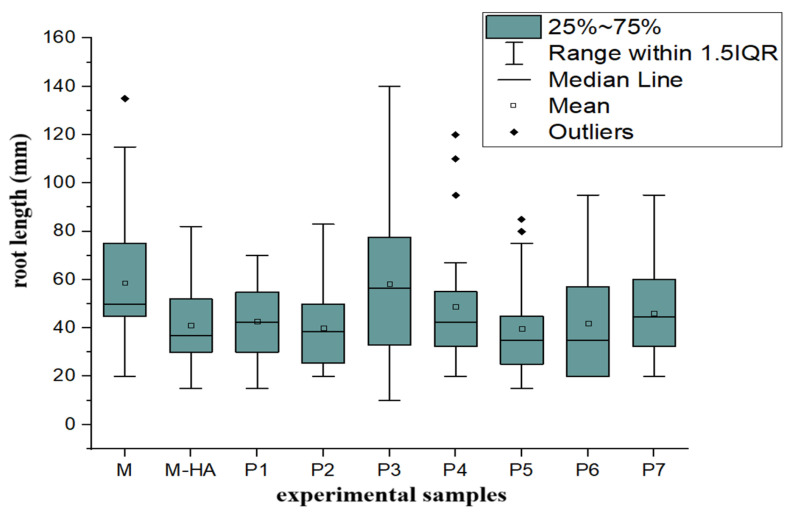
Box plot representation of the root lengths of seedlings developed in the presence of HA-MNP nanoparticles at different concentrations. M, control group treated with distilled water; M-HA, control group treated with 0.01% hyaluronic acid solution. P1 to P7 represent samples treated with different concentrations of HA-MNP: 0.625–1.25–1.875–2.5–3.125–3.75–7.5 mg/L (according to Table 2).

To evaluate the effect of HA-MNP on plant growth, the box plot method was applied to analyze the stem and root lengths across nine experimental samples. These samples were cultivated under varying HA-MNP concentrations for a 7-day period. The results are illustrated in Figure 1 and Figure 2.

As observed in the box plot diagram of stem length (Figure 1), the lengths of the box plots are relatively small, corresponding to close values within a relatively narrow range, thus indicating a small dispersion of values. However, several large outliers demonstrate drastic responses in some plants.

The comparison of the positions of the medians reveals a slight increase for samples P2 and P3 and a slight decrease for samples P6 and P7 relative to the control sample. An analysis of the median values of stem length indicated an increase for the samples treated with low concentrations of HA-MNP up to 2.5 mg/L (*p* < 0.05) and a decreasing trend for the samples treated with the highest concentration of nanoparticles, 7.5 mg/L.

With regard to roots, as observed in the box plot of root length (Figure 2), the plotted box lengths exhibit a greater magnitude, indicating higher dispersion for the root values of the plants in each sample. However, there was no significant variation between samples, with the exception of P3, treated with 1.875 mg/L HA-MNP, which demonstrated substantially higher dispersion compared to the other samples. For the roots, the number of outliers was minimal.

Upon comparison of the median positions and mean values, a decreasing trend in root length was evident for all samples, except sample P3, treated with 1.875 mg/L HA-MNP, for which the median value exceeded that of the control sample. In this case, the high values representing maxima and extreme values are absent, suggesting that this concentration of nanoparticles may result in enhanced rooting compared to the control sample. The differences in stem length (*p* < 0.05) and root length (*p* < 0.01) between the experimental groups were statistically significant according to one-way ANOVA analysis, with the differences in root length being more pronounced.

**Figure 3:**
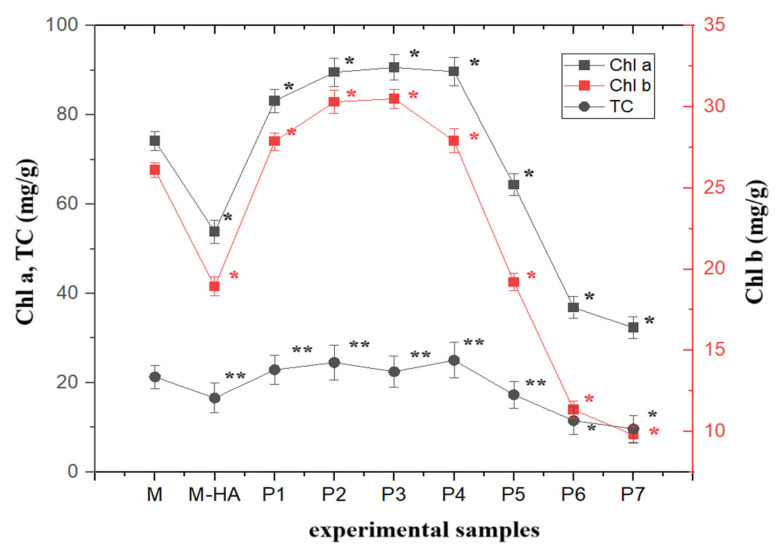
Photo-assimilatory pigment concentrations of the experimental samples (Chl a is the concentration of chlorophyll a in plant fresh tissue, Chl b is the concentration of chlorophyll b in plant fresh tissue and TC is the concentration of total carotenoids in plant fresh tissue). M, control group treated with distilled water; M-HA, control group treated with 0.01% hyaluronic acid solution. P1 to P7 represent samples treated with different concentrations of HA-MNP: 0.625–1.25–1.875–2.5–3.125–3.75–7.5 mg/L (according to Table 2). Data are expressed as means ± standard deviation. * Statistically significant differences (*p* < 0.05); ** statistically no significant differences (*p* > 0.05).

For the sample treated with 0.01% hyaluronic acid solution only (second control) (M-HA), a negative influence on both stem and root length was observed (both their mean and median values were lower than those for the control treated with distilled water only). According to the graphical representations of the level of the photo-assimilatory pigments of fresh tissues (Figure 3), the concentration of chlorophyll a (Chl a) showed a statistically significant increase of up to 22% in the samples treated with low concentrations of HA-MNP, up to 2.5 mg/L (*p* < 0.05), compared to that of the control sample. For nanoparticle concentrations higher than 2.5 mg/L, a statistically significant decrease (*p* < 0.05) of up to 56% was observed for the highest nanoparticle concentration tested in the experiment (7.5 mg/L).

A similar trend of variation was observed for the contents of the two types of chlorophyll, a and b; a linear correlation (Chl a = 2.83 × Chl b + 5.89) was observed between the levels of chlorophyll a (Chl a) and chlorophyll b (Chl b) for the nanoparticle-treated samples, with a correlation coefficient of R^2^ = 0.98. For chlorophyll b (Chl b), the increase was up to 16.7%, and the decrease was up to 37.4%. A comparable response was also observed for the total carotenoids pigment content, which exhibited an increase of up to 17.4% for low nanoparticle concentrations (<2.5 mg/L), and a decrease for concentrations exceeding 2.5 mg/L, reaching a maximum reduction of 45.3% at the highest concentration tested (7.5 mg/L).

A dose-dependent increase of up to 17.6% was observed in the chlorophyll ratio (Chl a/Chl b) relative to the control sample. For the second control treated with HA alone, no statistically significant alteration in the chlorophyll ratio was detected compared to the control sample. Furthermore, the experimental results demonstrated a significantly higher chlorophyll stability index (CSI) for samples treated with low concentrations of HA-MNP (samples P1, P2, P3 and P4) (up to 20%) and a significantly lower CSI for high concentrations of HA-MNP (samples P5–P7) (up to 42%) compared to the second control sample (M-HA).

For the greenness indicator, both elevated values (maximum 5.38 in sample P3) and reduced values (minimum 4.35 in sample P7, and 4.38 in sample M-HA) were observed in comparison to the corresponding control value (4.70). The obtained values fell within the normal range for this indicator [20], demonstrating that there were no adverse effects on the photosynthetic system of the plants. In plants exposed to sunlight, the greenness indicator typically ranges from 4.2 to 5, whereas in shade-exposed plants, it usually falls between 5.5 and 7.0 [20]. An elevated value in this indicator suggests a healthy plant with robust photosynthetic capacity and minimal stress, whereas a lower ratio may indicate adaptation to stress conditions, wherein carotenoids are present in higher proportions to protect plant cells from oxidative damage.

**Figure 4:**
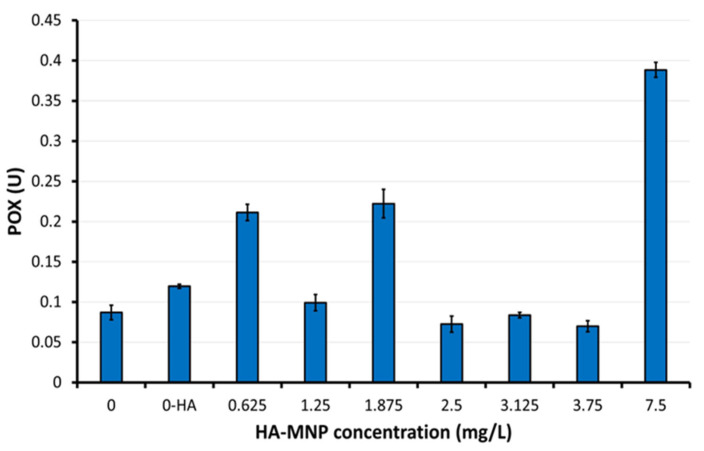
Peroxidase level (POX) of aerial parts of maize seedlings. 0-HA concentration corresponding to the second control sample, in which the seeds were treated with a 0.01% hyaluronic acid solution (M-HA). Data are means ± standard deviation.

Regarding the plant response in terms of antioxidant enzymes, based on the results obtained for the two types of enzymes analyzed in this study on aerial parts of maize seedlings, higher values of the peroxidase (POX) were observed in the second control (M-HA) compared to the first control, and in the sample treated with the highest concentration of nanoparticles (P7) in comparison to both control samples and to samples of lower concentrations of HA-MNP (Figure 4). For samples P4, P5, and P6, lower values were obtained compared to the corresponding control. The highest peroxidase activity was observed in the P7 sample, exhibiting a 4.46-fold increase compared to the corresponding control value. Elevated POX activity facilitates the elimination of excess reactive oxygen species (ROS).

**Figure 5:**
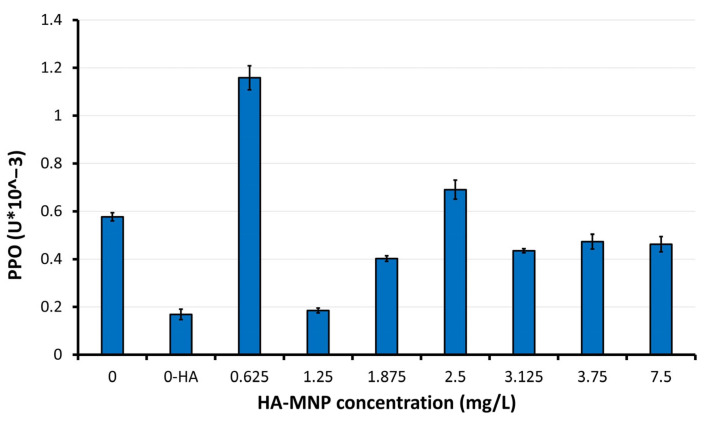
Polyphenol oxidase level (PPO) of aerial parts of maize seedlings. 0-HA concentration corresponding to the second control sample in which the seeds were treated with a 0.01% hyaluronic acid solution (M-HA).

The Kruskal–Wallis analysis applied for the POX mean values showed statistically significant differences between the samples treated with HA-MNP, in particular at higher and lower concentrations, and the non-treated samples/controls (*p* = 0.0017). The highest POX was registered in the sample treated with the highest HA-MNP concentration (7.5 mg/L). The lowest POX was found in samples treated with HA-MNP at 2.5 and 3.75 mg/L, respectively. The applied Dunn’s test confirmed statistically significant differences in POX between the 7.5 mg/L concentration and concentrations of 2.5 mg/L (*p* = 0.0149)/3.75 mg/L (*p* = 0.0085). Within the series of HA-MNP concentrations tested here, closer to the lower range, our results indicated a high POX at 1.875 mg/L, similar to that of the lowest concentration (0.625 mg/L) value, which was found to be marginally significantly higher than that of 3.75 mg/L (*p* = 0.0607). No statistically significant differences in POX were found among the other applied concentrations. Regarding the polyphenol oxidase (PPO) (Figure 5), increased values relative to the control sample were observed in samples P1 and P4, while all other samples exhibited reduced PPO activity. The maximum PPO activity was observed in sample P1 at the lowest HA-MNP concentration 0.625 mg/L, demonstrating a 2-fold increase compared to the value of first control sample, whereas the minimum value was obtained in the P2 sample at HA-MNP concentration 1.25 mg/L, showing a 3.6-fold decrease relative to the corresponding first control values.

The Kruskal–Wallis analysis (*p* = 0.0014) and the Dunn’s test showed that the PPO mean values were significantly lower in the control 0-HA than those in samples treated at 0.625 mg/L and 2.5 mg/L, respectively (*p* = 0.0057, and 0.0307, respectively). The highest PPO was registered at the lowest applied concentration (0.625 mg/L), with the results being statistically significant compared to the lowest PPO obtained in treated sample at 1.25 mg/L (*p* = 0.0149). A marginally significantly lower difference was observed between concentrations of 1.25 mg/L and 2.5 mg/L (*p* = 0.0715). The observed increase in PPO activity may indicate an enhanced phenolic metabolism [21], which serves as an indicator of the physiological adaptation of the plant to the presence of nanoparticles. This adaptation may occur either as a response to perceived stressors [22] or as an attempt to modify the phenolic metabolism to mitigate the oxidative damage caused by the presence of nanoparticles. Iron oxide nanoparticles have the potential to release iron ions, which are essential for various biological processes, including PPO activity [23]. Additional iron absorption could potentially stimulate the production of iron-dependent enzymes, thereby increasing the PPO activity without necessarily indicating stress. In the event that iron oxide nanoparticles stimulate other prioritized metabolic processes (such as photosynthesis through enhanced iron uptake), the plant may allocate metabolic resources to alternative pathways. Consequently, PPO activity might decrease because of the plant’s reduced perception of the acute need for phenolic defense.

**Figure 6:**
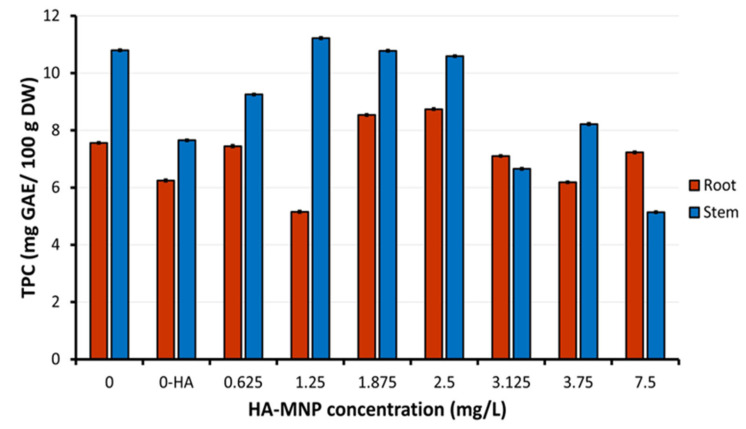
Total phenolic content (TPC) of aerial parts of maize seedlings. 0-HA concentration corresponding to the second control sample in which the seeds were treated with a 0.01% hyaluronic acid solution (M-HA). Data are means ± standard deviation.

Figure 6 illustrates the levels of total phenolic compounds (TPC) in the roots and stems of maize seedlings cultivated in the presence of varying concentrations of HA-MNP. At low nanoparticles concentrations (<2.5 mg/L), regarding those used in the treatment of seeds and resultant seedlings, a differential distribution of the TPC was observed. This distribution manifested as increased levels in stems and decreased levels in roots, or vice versa, compared to the control samples. This phenomenon suggests an adaptive metabolic redistribution of phenolic resources in accordance with the specific physiological requirements of each organ, which is influenced by the presence of nanoparticles in the culture substrate. This variability may indicate that the plant prioritizes either the protection of the organ directly exposed to nanoparticles (such as the roots) or defense and growth support of aerial structures (stems), thus reflecting differentiated responses to abiotic stress.

In contrast, at higher HA-MNP concentrations (>2.5 mg/L), a decrease in TPC was observed in both organs (stems and roots) compared to the level obtained in control samples. The decrease in TPC in both organs may indicate either a reduction in polyphenol synthesis, potentially due to the interference of nanoparticles with the metabolic processes involved, or an increased consumption of polyphenols in other physiological processes, such as in neutralizing oxidative stress or reinforcing the cell structure. Nanomaterials can influence secondary metabolism in plants [24]. Compounds resulting from plant secondary metabolism, including phenolic compounds, play a significant role in plant defense mechanisms against biotic and abiotic stressors. Zhang et al. (2020) demonstrated that the antioxidant activity of maize seeds is positively associated with the total phenolic compound content [25]. Notably, the total phenolic compound content in the secondary control sample (M-HA) treated only with the nanoparticle stabilizer, hyaluronic acid, also exhibited a decrease in the TPC in both organs (stems and roots) compared to that in the control sample.

The Kruskal–Wallis analysis showed highly significant differences in mean TPC values between control and samples treated with HA-MNP at different concentrations, both in stems (*p* = 0.001175) and roots (*p* = 0.001172). Significant differences were found in roots treated with HA-MNP, between concentrations of 1.25 mg/L and 1.875 or 2.5 mg/L, between 2.5 mg/L and 3.75 mg/L. The TPC in stems showed significant differences between first control sample and samples treated with 7.5 mg/L HA-MNP, between 1.25 mg/L and 3.125 or 7.5 mg/L, respectively.

**Figure 7:**
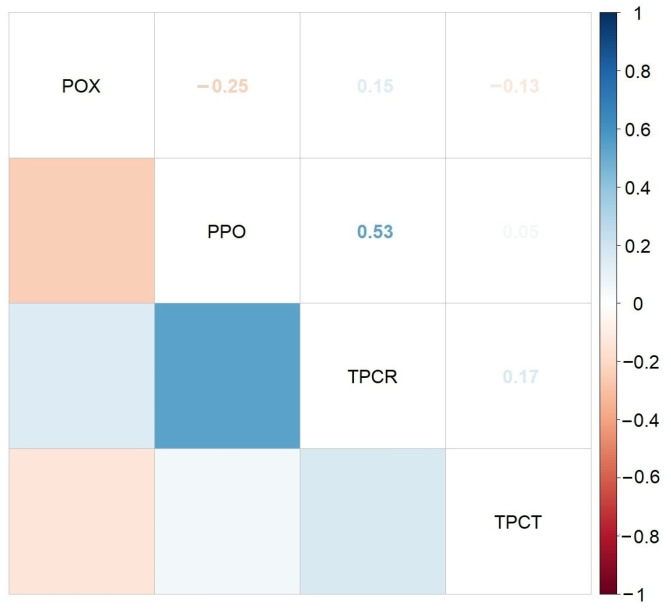
Correlation plot of Spearman’s correlation coefficients between enzymatic activities and TPC in investigated maize samples; TPCR = TPC in roots; TPCT = TPC in stems.

The Spearman correlogram presented in Figure 7 indicates that there were no statistically significant correlations between enzymatic activity (POX, PPO) and phenolics in stems and roots of maize seedling treated with different concentrations of HA-MNP. However, the greatest Spearman’s correlation coefficient value was found between PPO activity and TPC in roots (r = 0.53, *p* = 0.1392).

### 2.2. HA-MNP Genotoxicity on Maize

**Figure 8:**
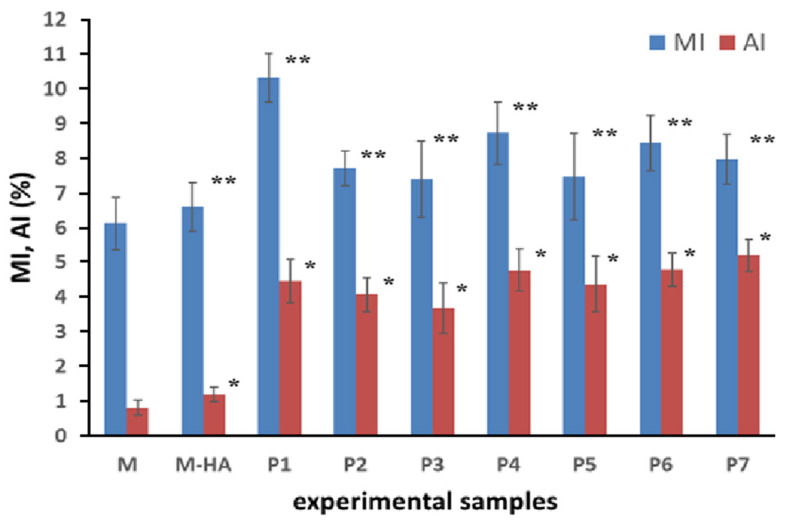
Mitotic index (MI) and aberrations index (AI) in germinated maize seeds treated with different HA-MNP concentrations (mg/L). Data are means ± standard deviation. * Statistically significant differences (*p* < 0.01), ** Statistically significant differences (*p* < 0.05).

Figure 8 displays the findings for both cytogenetic indicators of interest in the genotoxicity study: the mitotic index (MI) and the aberration index (AI). The MI values for all HA-MNP concentrations applied in maize treatment were significantly higher than those of the controls, suggesting that the HA-MNP suspension, when used as a seed germination substrate, could alter mitotic division in root tip cells.

The most pronounced increase in the MI was observed at the lowest HA-MNP concentration (0.625 mg/L). At higher HA-MNP concentrations, the MI variations from the control were less substantial. The highest HA-MNP concentration used in the present study showed a downward trend in MI compared to the lower concentrations, although it still exceeded the value of the control sample. Statistical analysis revealed that all cytogenetic results (MI and AI values) of the treated samples differed significantly from those of the control (*p* < 0.05). Statistical analysis of both the MI and AI indices revealed a statistically significant difference between the sets of values. This indicated that the presence of HA-MNP in the germination medium significantly affected mitotic division in the meristematic tissue of maize seeds. Additionally, HA-MNP influenced the occurrence of chromosomal aberrations at the cellular level, which was relatively low, with a maximum of 5.2% (Figure 8). A significant increase in the percentage of abnormal cells (AI) was recorded for all HA-MNP-treated samples compared to the control group (up to 84%). It can be observed that the number of chromosomal aberrations was higher in the sample treated with the highest HA-MNP concentration (7.5 mg/L) than that for the other samples. Statistical analysis revealed a significant positive relationship between cytogenetic indices (MI and AI). This was evidenced by Kendall’s tau correlation coefficient of 0.688 (*p* = 0.002) and Pearson’s coefficient of 0.799 (*p* = 0.001). Additionally, a direct linear relationship was identified between the mitotic index and seedling length. This correlation indicated a strong link between cellular division and plant growth when exposed to HA-MNP. Statistical analysis revealed a Pearson’s correlation coefficient of 0.91 (*p* < 0.001) between MI values and stem lengths. The observed correlation suggests that higher cell division rates are associated with accelerated seedling growth.

**Figure 9:**
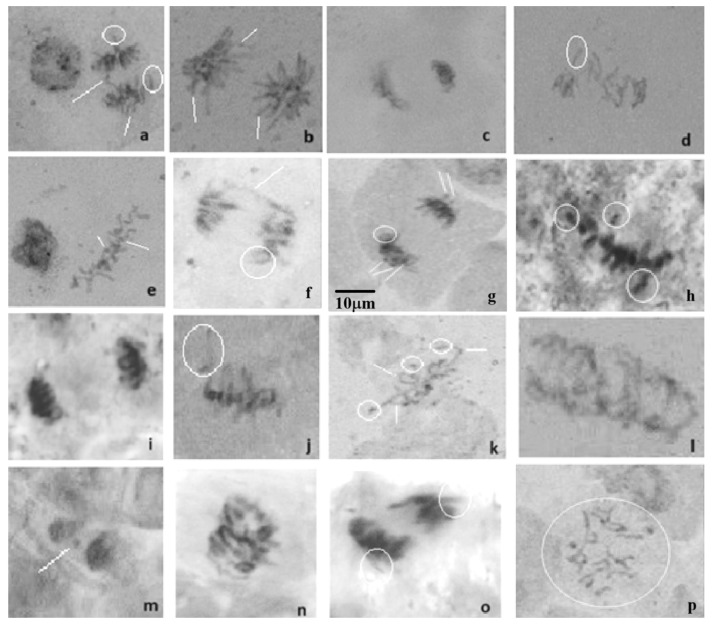
Microscopic examination revealed notable abnormal cells, as shown in photomicrographs: (**a**) anaphase with vagrant chromosomes; (**b**) anaphase with star effect and chromosome gaps; (**c**) asymmetric telophase; (**d**,**j**) metaphase with laggard chromosomes; (**e**) metaphase with chromosomal fragments; (**f**) anaphase with interchromatin bridge and ring chromosome; (**g**) anaphase with ring chromosome and micronucleus; (**h**) sticky metaphase with chromosomal fragments; (**i**) sticky anaphase; (**k**) metaphase with laggard chromosomes, ring chromosome, and fragments; (**l**) multi-polar delayed anaphase; (**m**) binucleated cell with micronucleus; (**n**) delayed anaphase; (**o**) sticky telophase with vagrant chromosomes; (**p**) C-mitosis. Specific chromosomal aberrations are highlighted using circles and lines in the images.

Chromosomal aberrations were identified based on the established scientific literature [26,27] and our prior research findings [28,29]. Photomicrographs of the abnormal cells observed during microscopic analysis are shown in Figure 9. Laggard and vagrant chromosomes in metaphase and anaphase, respectively, were some of the main types of abnormal cells observed in the present experimental study. These abnormal cell types are known to be physiological aberrations. It seems that the presence of HA-MNP in the seed germination substrate may influence centromere division. The additional common aberrations observed in this study were C-mitosis and stickiness. Chromosome stickiness is often indicative of toxic effects that may result in cell death [30]. The observation of C-mitosis in this study could be attributed to the impact of HA-MNP on the spindle apparatus, causing chromosomes to scatter randomly within the cells. C-mitosis can lead to numerous chromosomal aberrations, including polyploidy and aneuploidy [30].

Table 1 presents the types of abnormal cells observed in the root meristems treated with HA-MNP.

## 3. Discussion

Our study investigated how maize seedling growth was influenced by different concentrations of HA-MNP suspension used as growth media. The presence of low concentrations (<2.5 mg/L) of the HA-MNP nanoparticles used in this study appear to have a beneficial effect on maize plants, stimulating growth and metabolism without inducing significant stress. These positive effects could be attributed to enhanced iron uptake facilitated by the presence of iron oxide nanoparticles in the growth substrate, which is crucial for chlorophyll synthesis and overall plant health. Increased chlorophyll and carotenoid levels indicate enhanced photosynthetic activity, whereas improved antioxidant enzyme responses suggest efficient management of oxidative stress.

The results obtained for the hyaluronic acid-treated sample (M-HA), used as second control, suggested that HA alone induced metabolic stress in maize plants. This was evident from the reduced PPO activity, increased POX activity, reduced TPC in stems and roots, reduced level of chlorophylls and carotenoids, and reduced stem and root lengths compared to the first control sample. Decreased PPO activity, associated with reduced TPC, indicates a depletion of phenolic resources required for defense, whereas increased POX reflects a compensatory antioxidant response to neutralize oxidative stress. The POX activity is related to minimization of the impact of reactive oxygen species produced under stress conditions, but also to cell wall modification processes [31]. Other results showed that stress induced by chromium applied to the maize seed growth medium led to increased POX activity as a defense mechanism [32]. Decreases in PPO activity with the intensification of a stressor have previously been reported in the literature [33]. Moreover, it is considered that PPO activity influences plant vigor, and there is a hypothesis that requires further investigation, according to which the enzyme plays a protective role on the structures involved in photosynthesis, during the actions of an abiotic stress factor [34]. The presence of phenolic substrates influences the PPO activity, and the appearance of a stress factor could activate the latent form of the enzyme [22].

Higher concentrations of HA-MNP (>2.5 mg/L) exhibited an overall negative effect of nanoparticles on the physiological and biochemical parameters of the treated plants, suggesting potential toxicity. The decrease in PPO and POX activities, respectively (except at the highest concentration when it was increased) reflects an alteration in the antioxidant response and oxidative defense mechanisms of the plants, which may be associated with oxidative stress. In addition, lower chlorophyll and carotenoid concentrations in treated samples compared to the control indicate an inhibition of photosynthesis. The reduction in plant and root lengths points to a direct impact on growth and development, possibly as a result of stress accumulation or interference of nanoparticles with essential physiological processes. Taken together, these changes suggest that concentrations greater than 2.5 mg/L of the tested nanoparticles cause abiotic stress, negatively affecting the growth of maize. Given that the hyaluronic acid solution produces effects similar to those of hyaluronic acid-stabilized nanoparticles for higher concentrations, it is likely that the influence of the stabilizer is a major factor in determining the negative response, especially at high concentrations.

Our results are in agreement with findings from other studies. Plaksenkova et al. (2019) observed that low concentrations of iron oxide nanoparticles (1, 2, and 4 mg/L) can promote the growth of rocket *Eruca sativa* and significantly increase the shoot lengths [35]. Comparable growth-promoting effects of iron oxide nanoparticles were noted in *Ocimum basilicum* seedlings, at doses of up to 3 mg/L enhancing seedling elongation [36]. Kokina et al. (2020) showed that introducing iron oxide nanoparticles at concentrations up to 4 mg/L improved the length of *Medicago sativa* seedlings [37]. Wang et al. (2019) indicated that iron oxide nanoparticles enhanced *Curcumis melo* plant growth [38]. Li et al. (2016) observed that in *Zea mays*, iron oxide nanoparticles at 20 mg/L boosted the germination index by 27.2% and root elongation by 11.5%, but higher concentrations up to 100 mg/L significantly reduced root length to 13.6% and chlorophyll content to 39.9% [39]. Souza et al. (2019) noted that 10 mg/L of iron oxide nanoparticles increased chlorophyll a and b in *Lemna minor* [40]. Iannone et al. (2016) examined the physiological effects of citric acid-stabilized iron oxide nanoparticles on *Triticum aestivum L.* in hydroponic conditions, finding that a 10 mg/L concentration improved root length and germination [41]. Iannone et al. (2021) revealed increased chlorophyll levels and catalase (CAT) activity in soybean and alfalfa seedlings when treated with citric acid-stabilized iron oxide nanoparticles at doses up to 100 mg/L [42]. A significant increase in CAT activity was also noted in ryegrass and pumpkin seedlings exposed to polyvinylpyrrolidone-coated iron oxide nanoparticles at similar concentrations [43]. Hu et al. (2017) reported enhanced CAT and POD activities in *Citrus maxima* seedlings subjected to iron oxide nanoparticle treatment at concentrations reaching 100 mg/L, compared to the control group [4]. According to Li et al. (2013), watermelon plants exhibited substantially higher CAT and POD activities when exposed to iron oxide nanoparticles at concentrations of up to 50 mg/L [44]. Additionally, iron oxide nanoparticles at concentrations not exceeding 50 mg/L were found to boost the chlorophyll content in *Pseudostellaria heterophylla* plants [45].

In contrast, Martínez-Fernández et al. (2016) found that exposing *Helianthus annus* to iron oxide nanoparticles at 50 and 100 mg/L in a hydroponic culture led to decreased root length and nutrient uptake [46]. Similarly, Ding et al. (2019) reported that 200 mg/L of iron oxide nanoparticles significantly reduced the chlorophyll content and CAT enzyme activity in the aquatic plant *Eichhornia crassipes* [47].

Regarding oxidative stress and antioxidant responses, previous studies have indicated that nanoparticle exposure can modulate polyphenol accumulation, secondary metabolite production, and enzyme activity involved in oxidative protection [48]. Taghizadeh et al. reported a significant enhancement in the total phenolic content of *Dracocephalum polychaetum Bornm* suspension cultures exposed to iron oxide nanoparticles [49]. *Carum copticum L.* seedlings treated with 200 mg/L iron oxide nanoparticles had the highest total phenolic content, showing an increase of approximately 259% compared to the control group [5]. However, treatment with 100 and 400 mg/L iron oxide nanoparticles did not lead to any notable increase in the phenolic content [49]. In contrast, a study by Javed et al. (2017) on *Stevia rebaudiana* revealed that zinc oxide nanoparticles at concentrations of 100 and 1000 mg/L significantly decreased phenolic concentration [50].

In terms of genotoxicity, our results indicate that HA-MNP enhanced mitotic division activity, with a concentration-dependent increase in the mitotic index up to 7.5 mg/L. This is consistent with previous reports showing that iron oxide nanoparticles influence the mitotic index and chromosomal aberrations in wheat (Triticum aestivum) [51], supporting the idea that nanoparticle type, stabilizing agents, and plant species modulate genotoxic effects. A study conducted by Giorgetti et al. (2011) on Daucus carota exposed to iron oxide nanoparticles demonstrated a decreased mitotic index from 5.2% for the control sample to 2.5% in samples treated with a 6.7 mg/L concentration [52]. Singh et al. (2022) observed a reduced mitotic index in Allium cepa treated with iron oxide nanoparticles at concentrations up to 20 mg/L [53]. Gantayat et al. (2020) have indicated a potential genotoxic effect of iron oxide nanoparticles at concentrations up to 100 mg/L on Allium cepa [54]. The interaction between iron oxide nanoparticles and cell nuclei involves several steps. Initially, the nanoparticle suspension is absorbed by the roots and diffuses into the cell cytoplasm. Iron ions are then released from the nanoparticle surface and enter the cell nucleus, where they are believed to trigger a catalytic process. This process may lead to the breakdown of water molecules through iron-mediated Fenton reactions, resulting in the production of free water radicals. These radicals can have harmful effects, particularly on chromosome behavior during cell division. Consequently, abnormal mitosis may occur, potentially leading to genetic mutations. While cellular defense mechanisms may counteract some of these effects, others are likely to persist as permanent genetic alterations.

The observed effects of HA-MNP on maize seedlings suggest a complex toxicity mechanism involving both stimulatory and inhibitory responses, depending on nanoparticle concentration. The slight increase in shoot/root length and chlorophyll content at lower concentrations suggests that HA-MNPs may enhance growth by improving iron bioavailability or by influencing cellular metabolism. However, at higher concentrations, mild inhibitory effects were observed, possibly because of oxidative stress or nanoparticle accumulation in plant tissues. The enzymatic activities of POX and PPO, along with total polyphenol levels, displayed nonlinear trends, suggesting that oxidative stress modulation may depend on both HA-MNP concentration and plants’ adaptive responses. While the mitotic index increased at all tested concentrations, indicating enhanced cell division, the slightly elevated frequency of chromosomal aberrations (≤5.2%) suggests potential genotoxic effects, likely due to reactive oxygen species (ROS)-mediated DNA damage or interactions with mitotic spindle components. These findings align with those of previous studies reporting dose-dependent nanoparticle interactions with plant cells, where lower concentrations may act as stimulants, while higher doses induce oxidative stress and genomic instability. Therefore, the toxicity mechanism of HA-MNP in maize seedlings appears to be multifactorial, involving (i) concentration-dependent modulation of growth and physiological parameters, (ii) oxidative stress-related enzymatic activity shifts, and (iii) increased mitotic activity alongside minor chromosomal instability.

Overall, our findings contribute to a better understanding of the effect of HA-MNP on maize physiology and development. They suggested that, while low concentrations may offer potential benefits by enhancing iron uptake and antioxidant responses, higher concentrations can induce oxidative stress and impair growth. These results highlight the importance of optimizing nanoparticle concentrations for agricultural applications to balance beneficial effects with potential toxicity.

## 4. Materials and Methods

### 4.1. Experimental Design

For this investigation, a first control sample was prepared by treating the seeds with distilled water only (M), a second control sample by treating the seeds with a 0.01% hyaluronic acid solution (M-HA), and seven samples by treating the seeds with solutions of different concentrations of nanoparticles (P1-P7) (Table 2). The hyaluronic acid-stabilized nanoparticle sample (HA-MNP), with an average size of nanoparticles about 9.05 ± 2.89 nm, sizes mostly between 3 and 20 nm, and a globular shape [55], was used for seed treatments. The saturation magnetization of the HA-MNP sample used in this study was about 57.29 emu/g [55]. The concentrations of HA-MNPs used in this study were selected based on the results of similar experiments reported in the literature and the results of our preliminary investigations. Initially, volumetric fractions of the nanoparticle suspensions were chosen to test their potential effects on plant growth with increasing concentrations to explore the threshold at which toxicity might occur. The highest volumetric fraction (600 µL/L) was used to investigate the possible toxic effects, following a common approach in nanoparticle-related research. Table 2 lists the corresponding nanoparticle concentrations for each volumetric fraction used in the experiment. This approach ensured a broad range of effects, from beneficial to potentially toxic effects, and provided a comprehensive analysis of the impact of HA-MNPs on maize seedlings. To evaluate the independent effects of hyaluronic acid (HA), a control treatment utilizing 0.01% HA solution (without nanoparticles) (M-HA) was included. This concentration was selected based on its reported safety and non-toxic effects in previous plant studies [56], ensuring that any observed effects on plant growth could be attributed to nanoparticles rather than to HA itself. For each experimental sample, two replicates were prepared, with each replicate consisting of 25 seeds germinated in 15 mL of the corresponding liquid. Aqueous suspensions of HA-MNP were freshly prepared from the original nanoparticle sample at the amount required for each addition to the culture medium to prevent oxidation. Under these conditions, the nanoparticles were expected to remain stable in the suspension during the experimental period.

The biological material chosen for this research was *Zea mays* (maize) seeds, owing to their considerable economic significance in both the agricultural and food industries. An experimental set of intact maize seeds, characterized by consistent genetic traits and free from visible imperfections, insect-related damage, or deformities, was procured from a local cultivator in Săliște, Sibiu County, Romania, where the maize was cultivated indigenously. The seeds were sterilized the day prior to germination with 70% ethyl alcohol for 2 min and 2% *v*/*v* sodium hypochlorite, followed by three rinses with distilled water, and then dried. Maize caryopses, intact and uniform in both size and coloration, lacking wrinkles, underwent germination in Petri dishes. The germination medium consisted of a filter paper moistened with an appropriate liquid solution. Seven different volume fractions of HA-MNPs aqueous suspensions were used (50, 100, 150, 200, 250, 300 and 600 µL/L) (Table 2). Each sample received 15 mL of HA-MNP solution at the appropriate concentration as the culture substrate. To evaluate the impact of the magnetic nanoparticle stabilizer on germination and cell division, a separate sample was treated with a 0.01% hyaluronic acid solution in water (15 mL), serving as a second control. The germination process was conducted in a laboratory room under rigorously controlled environmental parameters, including complete absence of light and a constant temperature of 25 ± 0.5 °C.

Maize seedlings were cultivated under controlled environmental conditions: a temperature of 26.0 ± 0.5 °C, a light/dark cycle of 12 h light and 12 h dark, and 55% air humidity, without air currents, in a laboratory room. Samples were supplied with 15 mL of an HA-MNP aqueous suspension at a specific concentration daily at 8 a.m. for 7 days post-germination. The first and second control samples were subjected to identical environmental conditions, receiving only an equivalent volume of deionized water and 0.01% hyaluronic acid solution, respectively.

### 4.2. Assay of Genotoxicity

For cytogenetic analysis, germinated seeds aged two–three days with 1–2 cm root lengths were chosen. The root tips were preserved in Carnoy’s solution (glacial acetic acid/absolute ethanol; 1/3, *v*/*v*) for 24 h and then stored in 70% ethanol at 4 °C. The staining process involved soaking root tips in a 37% HCl/distilled water (1/1) solution for 25 min, followed by storage in modified carbol-fuchsin dye [57] in a refrigerator. Five microscope slides were prepared for each variant using the squash technique [58], with individual root tips from each germinated seed crushed on a slide in 45% acetic acid [59]. The mitotic index (MI, %) and chromosomal aberration index (AI, %) were calculated using the formulae of Bakare et al. (2000) [60] following cytogenetic analysis. The Euromex IS 1153-EPL optical microscope (40× objective, Euromex Optics, Arnhem, The Netherlands) with an attached CMEX-18000-PRO digital camera (Euromex Optics, Arnhem, The Netherlands) and Euromex ImageFocus Alpha software (vers. x64) were employed to identify cells at various mitotic division stages and chromosomal aberrations. The same operator analyzed at least 2000 cells and over 30 microscopic fields for each microscope slide.

### 4.3. Assay of Phytotoxicity

Germination seed percentage was calculated according to the formula described by Dehnavi et al. (2020) [61]. Measurements of the length of the stems as well as of the roots, which were taken after seven days of growth, were carried out carefully using a ruler with an accuracy of 1 mm. Using a MAC210 infrared thermobalance with 10^−3^% precision, the water content of the fresh tissue was measured at 105 °C.

The effects of HA-MNP on chlorophyll and carotenoid levels at various nanoparticle concentrations in culture media were determined. In the presence of low quantities of MgCO_3_ and CaCO_3_, fresh tissue (approximately 0.2 g) from each sample was macerated and homogenized in 5 mL of 80% acetone (SILAL Trading, Chemical Reagent Company, Bucharest, Romania). The homogenate was centrifuged and filtered. The pigment extract was diluted to 10 mL using 80% acetone. The chlorophyll content in the fresh tissue was analyzed using a spectrophotometric method employing a Specord 200 Plus UV–VIS spectrophotometer (Analytik Jena, Jena, Germany) equipped with quartz cells 1 cm in width. Spectrophotometric measurements were conducted at wavelengths of 646.8 nm, 663.2 nm, and 470 nm [20]. Chlorophyll and total carotenoids levels were calculated according to Lichtenthaler’s Formulas (1)–(3) [20] and expressed in mg per gram fresh weight (mg/g):

Chl a = 12.25 A663.2 − 2.79 A646.8 (1)

Chl b = 21.50 A646.8 − 5.10 A6663.2 (2)

TC = (1000 A470 − 1.82 Chl a − 85.02 Chl b)/198(3)

where Chl a is the concentration of chlorophyll a in plant fresh tissue, Chl b is the concentration of chlorophyll b in plant fresh tissue and TC is the concentration of total carotenoids (sum of xanthophylls and carotenes) in plant fresh tissue.

The chlorophyll stability index (CSI) was also calculated, which provides a measure of plant resilience to stress conditions, according to the relationship given by Pandiyan et al. (2017) [62]. In addition, we determined a plant greening indicator [20], which is given by the ratio of the sum of chlorophyll content (Chl a + Chl b) to the total carotenoid pigment content (TC). This indicator is used to assess the health, vigor, and chlorophyll content of plants. It provides information on the physiological and metabolic status of plants and can be used to monitor plant growth, productivity, and responses to various environmental conditions.

### 4.4. Assay of Enzymes Activity

Peroxidase activity (POX) in maize extracts, obtained from aerial parts of the plant, was determined using PBS buffer at pH 6.5, following the methodology outlined by Reddy et al. (1995) [63]. The experimental procedure involved the addition of the extract to a solution containing 3 mL of 0.05 M pyrogallol and 0.5 mL H_2_O_2_. Absorbance changes were monitored at 430 nm using a Specord 200 Plus UV–VIS spectrophotometer (Analytik Jena, Jena, Germany). Enzyme activity was quantified in units, with one unit defined as the change in absorbance per minute at a specified wavelength.

For the assessment of polyphenol oxidase activity (PPO), maize extracts obtained from aerial parts of the plant were prepared in Tris-HCl buffer at pH 7.2, following the protocol described by Esterbauer et al. (1977) [64]. The experimental setup involved the combination of the extract with 0.3 mL (0.01 M catechol). Absorbance was measured at 495 nm using a spectrophotometer. In this case, enzyme activity was expressed in units, where one unit represented the quantity of enzyme capable of converting 1 μmol dihydrophenol to 1 μmol quinone per minute.

### 4.5. Assay of Phenolic Compounds

Plant samples (roots or stems) were subjected to ethanolic extraction using a solvent-to-solid ratio of 10:1. The total phenolic content (TPC) of the extract was quantified spectrophotometrically using the Folin–Ciocalteu methodology [65]. The measurements were conducted using a Specord 200 Plus UV-VIS spectrophotometer (Analytik Jena, Jena, Germany). The results were reported as mg gallic acid equivalents (GAE) per 100 g dry weight (DW) of the sample.

### 4.6. Statistical Analysis

Two replicates were prepared for each sample. All biochemical analyses were conducted in duplicates. Descriptive statistical parameters were calculated. The statistical significance between the control and treated samples was evaluated using the ANOVA single factor, with significance defined by a probability level of *p* < 0.05. Correlation analysis between different parameters was performed by calculating Kendall’s tau and Pearson’s correlation coefficients. Microsoft Excel 2016 and OriginLab software (ver. 2023b) were used to execute all statistical analyses and graphical representations. The differences between the mean values of TPC/POX/PPO at different HA-MNP concentrations were evaluated by the nonparametric Kruskal–Wallis test and Dunn’s test [66], using R software, version 4.4.1 [67]. A Spearman correlation analysis was performed on experimental data of TPC, POX, and PPO [68], with the results being presented in the form of a correlogram obtained using the function “corrplot.mixed” from corrplot package (version 0.94) [69].

## 5. Conclusions

Hyaluronic acid-stabilized iron oxide nanoparticles (HA-MNP) were applied at seven different concentrations (0.625–7.5 mg/L) to the growth medium of the maize seeds, which were monitored in parallel with two control samples. The use of nanoparticles concentrations up to 2.5 mg/L supported the growth and development of maize seedlings, maintaining the root moisture contents at values that were similar to control, ensuring a germination percentage of 100%, longer aerial parts, higher amounts of carotenoids and chlorophyll, and lower phenolic content (TPC) in the roots, but higher in the aerial parts, and strongly affecting the mitotic division of meristem cells. Concentrations higher than 2.5 mg/L decreased the level of carotenoids in the seedlings and the TPC in roots and stems. The maize treatment with 7.5 mg/L HA-MNP produced the highest number of chromosomal aberrations and the highest moisture content of the stems. The control sample tested in a medium containing 0.01% hyaluronic acid did not show considerable changes in chlorophyll a/chlorophyll b ratio, while samples exposed to HA-MNP showed a modified ratio, as well as the chlorophyll stability index, which increased at high concentrations. In contrast, our results showed that HA induced most of the abiotic stress on maize seedlings in samples with stabilized nanoparticles. The strongest activities of peroxidase and polyphenol oxidase were found at the highest HA-MNP concentration (7.5 mg/L) and, respectively, the lowest one (0.625 mg/L). Their lower values were associated with decreased TPC. HA-MNP significantly affected the cell division of root meristems in all samples, producing higher values in the mitotic index and increased chromosomal aberrations compared to the control. Among the identified genetic aberrations in the root cells are the appearance of laggard and vagrant chromosomes, and the presence of C-mitosis and stickiness, increasing the potential risk of polyploidy cases or cell death. Given their effects on cell division and plant growth, HA-MNP could be further investigated as potential bio-stimulants to improve seed germination and early development. However, owing to the observed genotoxic effects at higher concentrations, further studies are needed to establish safe and effective application protocols in agriculture.

## Figures and Tables

**Table 1:** Experimental results of cytogenetic analysis of germinated seeds in the presence of HA-stabilized nanoparticles (HA-MNP).

C (mg/L)	TC	TD	TA	Total Number of Cells with Chromosomal Aberrations											
				Mn	Vg	Lg	DA	Cm	Bg	Bk	Rc	As	St	Pn	Co
0	18,419	1129	149	18	32	5	0	13	7	1	1	23	5	1	43
0-HA	17,273	1140	206	22	37	2	2	32	4	11	2	46	6	14	28
0.625	12,596	1300	562	30	141	2	4	24	2	9	3	23	12	10	302
1.25	13,070	1008	532	40	133	6	3	12	6	2	5	25	13	2	285
1.875	12,312	911	454	30	184	2	2	17	5	10	7	25	6	0	166
2.5	11,034	962	527	53	142	13	8	9	8	2	1	24	5	1	261
3.125	11,172	835	489	53	101	2	2	9	2	0	1	27	6	2	284
3.75	11,479	968	551	44	150	9	11	3	2	1	3	32	4	3	289
7.5	11,774	938	612	34	137	2	3	1	2	0	5	19	3	1	405

Abbreviations: C, concentration of HA-MNP nanoparticles used in the treatment; TC, total number of cells analyzed in each treatment, thus totaling five slides for each sample; TA, total number of abnormal cells for each sample; TD, total number of cells undergoing mitotic division for each sample; Mn, micronuclei; Vg, vagrant chromosomes; Lg, laggard chromosomes; DA, delayed anaphase; Cm, C-mitosis; Bg, chromatid bridges; Bk, chromosome breaks, fragments; Rc, ring chromosomes; As, asymmetries; St, stickiness; Pn, polynucleate cells; Co, other abnormal cells and combinations of aberrations.

**Table 2:** The HA-MNP concentration of aqueous diluted suspension samples used for plant samples’ treatment.

HA-MNP Suspension Volume Fraction (µL/L)	50	100	150	200	250	300	600
HA-MNP Concentration (mg/L)	0.625	1.25	1.875	2.5	3.125	3.75	7.5

## Data Availability

All the data are contained within the article.
